# Risk factors for treatment delay in pulmonary tuberculosis in Recife, Brazil

**DOI:** 10.1186/1471-2458-5-25

**Published:** 2005-03-18

**Authors:** Martinho APS dos Santos, Maria FPM Albuquerque, Ricardo AA Ximenes, Norma LCL Lucena-Silva, Cynthia Braga, Antônio RL Campelo, Odimariles MS Dantas, Ulisses R Montarroyos, Wayner V Souza, Alexandre M Kawasaki, Laura C Rodrigues

**Affiliations:** 1 Departamento de Medicina Tropical, Universidade Federal de Pernambuco, Brazil; 2 Departamento de Medicina Clínica, Universidade Federal de Pernambuco, Brazil, and Centro de Pesquisas Aggeu Magalhães, Fundação Oswaldo Cruz, Brazil; 3 Centro de Pesquisas Aggeu Magalhães, Fundação Oswaldo Cruz, Brazil; 4 London School of Hygiene and Tropical Medicine, University of London, UK

## Abstract

**Background:**

Tuberculosis is still a great challenge to public health in Brazil and worldwide. Early detection followed by effective therapy is extremely important in controlling the disease. Recent studies have investigated reasons for delays in treatment, but there is no agreed definition of what constitutes an "acceptable" delay. This study investigates factors associated with total delay in treatment of tuberculosis.

**Methods:**

A cohort of adult cases of pulmonary tuberculosis diagnosed over a two-year period was studied. Patients were interviewed on entry, reporting the duration of symptoms before the start of treatment, and sputum and blood samples were collected. It was decided that sixty days was an acceptable total delay. Associations were investigated using univariable and multivariable analysis and the population attributable fraction was estimated.

**Results:**

Of 1105 patients, 62% had a delay of longer than 60 days. Age, sex, alcoholism and difficulty of access were not associated with delays, but associations were found in the case of unemployment, having given up smoking, having lost weight and being treated in two of the six health districts. The proportion attributable to: not being an ex-smoker was 31%; unemployment, 18%; weight loss, 12%, and going to the two worst health districts, 25%.

**Conclusion:**

In this urban area, delays seem to be related to unemployment and general attitudes towards health. Although they reflect the way health services are organized, delays are not associated with access to care.

## Background

Tuberculosis is still a great challenge for public health in Brazil and worldwide. In Recife, a large city in the Northeast Region of the country, the annual incidence of tuberculosis is high, about 100 per 100 000 inhabitants in 2003.

Early detection of infectious tuberculosis cases followed by effective treatment is extremely important in controlling the disease, mainly because it can be assumed that the time of infection is likely to pre-date the positive sputum test by some time [1]. Because of this, recent studies have investigated reasons for delays in treatment [2-8].

The starting point from which the delays are measured is uncertain and there is no agreed definition as to what constitutes an acceptable delay. The cut off point in studies of risk factors for an acceptable delay has been defined in two ways: either a panel of experts agrees on a reasonable period of time or, alternatively, the median delay in observed data is used. Panels of expert have agreed on an acceptable total delay of 30 days [9,10] or 60 days [11,12]. Ward *et al*. [13] have criticized this approach, suggesting that what is an acceptable delay should depend on the local health services and the local epidemiological situation, with a shorter delay to be expected when incidence is high.

Studies using the median observed total delay as the cut off point found medians ranging from 2 to 4 months [2,3,8,14-16]. Studies using the median patient delay found medians ranging from 1 to 4 months [2,3,7,13,9,17,10]. Studies restricted to smear-positive cases have tended to find lower median patient delays [8,16].

Risk factors identified in the literature as associated with long total delays before treatment include: those relating to perception and knowledge (inadequate knowledge about TB) [5,7,9], lack of perception of the need to seek health care [18]; those relating to the difficulty of access to health care (living in rural areas)[14], and distance from health care units [7,8]; those relating to the severity or specificity of symptoms (vagueness of symptoms, absence of haemoptysis [5,14,9,16], negative smears [2]; those relating to old age [2,13,14,16], female sex [11,6,19,20], ethnic group (white patients) [6] and alcoholism [8]. The only study of delay between the onset of symptoms and treatment in patients with pulmonary tuberculosis carried out in Brazil did not look at risk factors [21].

The aim of this study was to investigate biological, clinical, social, life-style and health-care factors associated with total treatment delay among cases of tuberculosis in residents of Recife, to identify the main constraints on early detection of patients and thus help with deciding which measures to use to decrease tuberculosis transmission in Recife.

## Methods

This was an observational study consisting of a cohort of cases of pulmonary TB, aged 18 years or more, diagnosed in Recife between May 2001 and May 2003. Case finding, mostly passive, is one of the main activities of the national tuberculosis control program (TCP). Decentralization has been underway since 2000, with the progressive transfer of activities from tuberculosis (TB) reference units to primary care units, as part of the Family Health Program (FHP) [22]. One of the activities of FHP staff is to detect people with respiratory symptoms (a cough persisting for three or more weeks) and to have their sputum examined.

Patients were invited to participate in the study when the decision to start treatment was taken, i.e. when the diagnosis of tuberculosis was given according to the criteria established by the Ministry of Health. After signing the informed consent form they were interviewed by trained assistant nurses using a standard questionnaire, and had sputum and blood samples collected for examination. Patients were asked how long ago they first showed symptoms of pulmonary tuberculosis. In this way, the total delay (from onset of symptoms to start of tuberculosis treatment) was ascertained directly from the patients. Patients were not aware if they had acceptable or unacceptable delay; thus, recall bias, if it occurred, was likely to be non-differential.

To define acceptable total delay we worked with two cut off points: sixty and ninety days. An analysis of risk factors for delays of ninety days or more (the median of our data) found essentially the same factors in the univariable analysis as with sixty days, but only smoking and loss of weight remained in the final multivariable model. We concluded that a cut off point of sixty days enables more potential public health interventions to be identified.

Exposures studied included biological factors (age and sex), alcohol consumption, social factors (employment status, marital status, literacy; numbers of dwellers in residence, and income of the head of the household), history of contact with other cases of tuberculosis (knowing somebody with TB, early contact with TB patient, and living in the same house as a TB patient), clinical factors (previous tuberculosis treatment, cough, weight loss, acid-fast bacilli smear result, HIV co-infection, and haemoptysis), the Health District in which unit of treatment is located, and access to health services (number of health units attended with this complaint before start of treatment; whether unit of treatment is the local health center and whether it is in the district of residence; whether the Family Health Program had been implemented in the area of residence).

The univariable OR of unacceptable treatment delay was estimated for biological factors, alcohol consumption, social factors, history of contact with other cases of tuberculosis, clinical factors, the health district in which treatment was carried out, and access to health services. The significance of these associations was tested using the chi-squared test and p-values were calculated. All variables with a univariable association with a p-value ≤ 0.20 were included in a multiple logistic regression analysis. Crude and adjusted ORs and 95% CIs were calculated. The attributable-risk percentages in the final model were calculated as , where RR was the adjusted OR for each variable.

## Results

Of 1126 cases of pulmonary TB in the study population, 21 (1.9 %) were excluded because of lack of information about the time of onset of symptoms, leaving 1105 patients for the study. The patients excluded were similar to those included in terms of characteristics identified as predictors of delay in the univariable analysis, with the exception of weight loss. The mean total delay was 142 days, the median 90 days (120 in men and 90 in women). Sixty-two percent of patients had a total delay of longer than 60 days (which we consider an unacceptable delay in this analysis). In 18%, the delay was longer than 181 days.

Tables 1 to 5 show the results of univariable analysis of risk factors for unacceptable total delay. No association with delay was found in the case of: sex, age, neither heavy nor light alcohol consumption (table 1), literacy, income of head of household, marital status of case, size of household (table 2); history of contact with a case of tuberculosis (table 3); any clinical factor other than weight loss (including smear-positive test result) (table 4); and any factor concerning access to health services, except for district of treatment (table 5).

**Table 1:** Biological factors, alcohol and tobacco consumption and total delay in pulmonary tuberculosis treatment. Recife, 2001–2003.

	Total delay in treatment						
Biological factors	Unacceptable (> 60 days)		Acceptable (≤ 60 days)		Crude OR	95% CI	P value
	N	%	N	%			
Sex							
Male	470	63.4	271	36.6	1.00	-	-
Female	216	59.3	148	40.7	1.18	0.91 – 1.53	0.1884
Total	686	-	419	-	-	-	-
Age (years)							0.1810
18–39	344	60.2	227	39.8	1.00	-	-
40–59	280	65.4	148	34.6	1.24	0.96 – 1.61	0.0949
≥ 60	62	58.5	44	41.5	0.92	0.61 – 1.41	0.7349
Total	686	-	419	-	-	-	-
Alcohol consumption							0.5575
None	338	60.7	219	39.3	1.00	-	-
Light drinker	214	61.5	134	38.5	1.03	0.78 – 1.36	0.8075
Heavy drinker	83	65.9	43	34.1	1.25	0.83 – 1.87	0.2800
Total	635	-	396	-	-	-	-
Cigarette smoking							0.0010
Never smoked	213	68.9	96	31.1	1.00	-	-
Given up smoking	297	56.7	227	43.3	0.58	0.43 – 0.79	0.0005
Ever smoked	175	65.3	93	34.7	0.84	0.59 – 1.20	0.3545
Total	685	-	416	-	-	-	-

**Table 2:** Socio-economic factors and total delay in pulmonary tuberculosis treatment. Recife, 2001–2003.

	Total delay in treatment						
Socio-economic factors	Unacceptable (> 60 days)		Acceptable (≤ 60 days)		Crude OR	95% CI	P value
	N	%	N	%			
Employment							
Employed	282	57.3	210	42.7	1.00	-	-
Unemployed	404	65.9	209	34.1	1.43	1.12 – 1.83	0.0035
Total	686	-	419	-	-	-	-
Literacy							
Yes	540	61.6	337	38.4	1.00	-	-
No	146	64.0	82	36.0	1.11	0.82 – 1.50	0.4951
Total	686	-	419	-	-	-	-
Income of Head of household (MW)							0.7356
≥ 5	22	59.5	15	40.5	1.00	-	-
2 to < 5	75	66.4	38	33.6	1.34	0.62 – 1.88	0.4461
1 to < 2	184	63.7	105	36.3	1.19	0.59 – 2.40	0.6176
< 1	327	61.5	205	38.5	1.08	0.55 – 2.14	0.8086
Total	608	-	363	-	-	-	-
Civil status							0.9600
Married	251	62.4	151	37.6	1.00	-	-
Divorced, widowed or separated	64	62.7	38	37.3	1.01	0.64 – 1.58	0.9544
Single	341	61.7	212	38.3	0.96	0.74 – 1.26	0.8078
Total	656	-	401	-	-	-	-
Number of individuals per Household							0.3897
1 – 2	160	65.3	85	34.7	1.00	-	-
3 – 5	345	60.2	228	39.8	0.80	0.59 – 1.09	0.1700
≥ 6	165	61.6	103	38.4	0.85	0.59 – 1.22	0.3803
Total	670	-	416	-	-	-	-

**Table 3:** Contact with tuberculosis and total delay in pulmonary tuberculosis treatment. Recife, 2001–2003.

	Total delay in treatment						
Contact history	Unacceptable (> 60 days)		Acceptable (≤ 60 days)		Crude OR	95% CI	P value
	N	%	N	%			
Knows someone with tuberculosis							
Yes	305	63.0	179	37.0	1.00	-	-
No	375	61.4	236	38.6	0.93	0.72 – 1.19	0.5782
Total	680	-	415	-	-	-	-
Recent contact with someone with tuberculosis							0.8554
< 1 year	151	63.4	87	36.6	1.00	-	-
≥ 1 year	128	61.8	79	38.2	0.93	0.63 – 1.37	0.7263
Not known	375	61.4	236	38.6	0.91	0.67 – 1.24	0.5769
Total	654	-	402	-	-	-	-
Household contact with a case of tuberculosis							
Yes	104	62.7	62	37.3	1.00	-	-
No	198	63.1	116	36.9	1.01	0.68 – 1.50	0.9301
Total	302	-	178	-	-	-	-

**Table 4:** Clinical features and symptoms and total delay in pulmonary tuberculosis treatment. Recife, 2001–2003.

	Total delay in treatment						
Clinical features and symptoms	Unacceptable (> 60 days)		Acceptable (≤ 60 days)		Crude OR	95% CI	P value
	N	%	N	%			
Previous TB treatment							
Yes	147	60.5	96	39.5	1.00	-	-
No	537	62.5	322	37.5	1.08	0.81 – 1.45	0.5666
Total	684	-	418	-	-	-	-
Productive cough							
Yes	418	62.4	252	37.6	1.00	-	-
No	259	61.1	165	38.9	0.94	0.73 – 1.21	0.6655
Total	686	-	419	-	-	-	-
Haemoptysis							
Yes	94	56.6	72	43.4	1.00	-	-
No	585	62.9	345	37.1	1.29	0.92 – 1.81	0.1256
Total	679	-	417	-	-	-	-
Loss of weight							
Yes	451	59.6	306	40.4	1.00	-	-
No	225	68.8	102	31.2	1.49	1.13 – 1.97	0.0041
Total	676	-	408	-	-	-	-
HIV co-infection							0.6964
Yes	219	63.8	124	36.2	1.00	-	-
No	18	64.3	10	35.7	0.98	0.43 – 2.19	0.9631
Not known	449	61.3	284	38.7	0.87	0.39 – 1.92	0.7467
Total	686	-	418	-	-	-	-
Acid fast bacilli in sputum							
Yes	477	64.1	267	35.9	1.00	-	-
No	102	63.4	59	36.6	0.96	0.67 – 1.37	0.8557
Total	579	-	326	-	-	-	-

**Table 5:** Access to health care and characteristics of health service (HS) and total delay in pulmonary tuberculosis treatment. Recife, 2001–2003.

	Total delay in treatment						
Access to health care	Unacceptable (> 60 days)		Acceptable (≤ 60 days)		Crude OR	95% CI	P value
	N	%	N	%			
Numbers of health units							
only 1	176	63.1	103	36.9	1.00	-	-
≥ 2	493	61.2	312	38.8	0.92	0.69 – 1.22	0.5859
Total	669	-	415	-	-	-	-
HS in same district of residence							
Yes	431	61.3	272	38.7	1.00	-	-
No	255	63.6	146	36.4	1.10	0.85 – 1.42	0.4523
Total	686	-	418	-	-	-	-
HS in neighborhood of residence							
Yes	111	63.4	64	36.6	1.00	-	-
No	575	61.9	354	38.1	0.93	0.67 – 1.30	0.7012
Total	686	-	418	-	-	-	-
Residence in areas of FHP visits							
Yes	258	60.4	169	39.6	1.00	-	-
No	406	63.4	234	36.6	1.13	0.88 – 1.46	0.3196
Total	664	-	403	-	-	-	-
Health District of treatment							0.0001
HD II	170	52.8	152	47.2	1.00	-	-
HD I	57	62.6	34	37.4	1.49	0.92 – 2.41	0.0960
HD III	139	72.8	52	27.2	2.39	1.62 – 3.51	0.0000
HD IV	111	61.7	69	38.3	1.43	0.99 – 2.08	0.0553
HD V	172	67.7	82	32.3	1.87	1.33 – 2.64	0.0003
HD VI	37	55.2	30	44.8	1.10	0.64 – 1.87	0.7170
Total	686	-	419	-	-	-	-

Table 6 presents the results of the multivariable analysis. All variables that were significant in the univariable analysis remained so in the multivariable analysis. The factors significantly associated with delay were: being unemployed (OR = 1.4; 95% CI: 1.09 – 1.81); not having weight loss as one of the symptoms (OR = 1.54; 95% CI: 1.16 – 2.04); and treatment in two of the six districts (OR = 2.34; 95% CI: 1.57 to 3.47 and OR = 1.92 95% CI: 1.35 to 2.73). The unacceptable delay attributable to each of these factors was: unemployment, 28.6%, not having weight loss, 35.1%, and being treated at health districts III and V, 57.3% and 47.9% respectively.

**Table 6:** Final model of characteristics of patients and health services and total delay in pulmonary tuberculosis treatment. Recife, 2001–2003.

	Adjusted OR	95% CI	P value
Employment			
Yes	1.00	-	-
No	1.40	1.09 – 1.81	0.0079
Loss of weight			
Yes	1.00	-	-
No	1.54	1.16 – 2.04	0.0028
Health District (HD)			0.0001
HD II	1.00	-	-
HD I	1.35	0.83 – 2.21	0.2189
HD III	2.34	1.57 – 3.47	0.0000
HD IV	1.39	0.95 – 2.04	0.0863
HD V	1.92	1.35 – 2.73	0.0002
HD VI	0.97	0.56 – 1.68	0.9395

* variables excluded from model: sex, age and haemoptysis because they were not statistically significant.

## Discussion

In our setting there was no statistically significant association between unacceptably long total delays and living in a different district from the unit of treatment, negative smears, older age, female sex and alcoholism. We found independent, statistically significant association with long delays and being unemployed, having loss of weight as one of the symptoms, and living in two of the six health districts.

One of the limitations of the study is that we were not able to separate patient delay from health services delay. It is, therefore, possible that some associations are specific to one of these kinds of delay.

Some of the associations absent (for example with being a heavy drinker) [8] may be due to methodological limitations. We did not use the CAGE standard questionnaire to ascertain alcoholism. Another limitation of the study of the impact of the Family Health Program is that we did not ask whether there was a visit from the FHP after the onset of the symptoms or even whether symptoms were addressed during a visit.

In contrast to results reported in the literature, it was found that a history of contact with a patient with tuberculosis did not decrease delays. We expected this to act as a proxy variable for knowledge about the disease [5,7,9]. However, none of the variables relating to previous contact with a patient with TB was associated with treatment delay. Again in contrast to other studies [2,7,16], being smear-positive was not associated with a shorter delay and most of those patients (64.1%) did not start early treatment and continued to contribute to transmission. The only symptom that was associated with shorter delays was loss of weight. Productive cough, even when associated with haemoptysis, did not lead to shorter delays in the population studied, in contrast to the findings of other authors [8,14,16]. Our findings, taken as a whole, may suggest that it is not specific knowledge of tuberculosis *per se*, but rather a general awareness of health and attitude towards prevention and early care that leads to shorter delays.

If one assumes that the risk of delay associated with literacy, age and sex is mediated by difficulty of access, one could say that, in this case, access to care does not appear to be a problem. In contrast to results reported in the literature [2,4,8,13-15], we found no association with age, sex or literacy. Associations have been reported between travelling time, distance to health service etc. [7,8,17] in the literature. In our study, factors directly relating to health care, such as whether the treatment unit was in the same neighborhood of residence, showed no association. This lack of association, however, could also be interpreted to mean that access is not difficult in this urban setting, but that ease of access did not decrease delays, as those attending health units outside their neighborhood or district of residence show the same length of delay. Decentralization has been introduced to improve access and we expected those patients being treated in their own neighborhood of residence to exhibit shorter delays. Another of the health service variables, however, did prove to be significant: patients attending health units located in districts III and V showed longer delays. This did not disappear in the multivariable analysis, suggesting that the delays were not a result of characteristics of patients but of the way the health service is organized. Further analysis (not presented here) showed that, in district V, this was mainly caused by delays in diagnosing smear-negative patients: only 10% had a delay of less than 60 days, compared with roughly 30% of the smear-positive cases. This is, therefore, more likely to reflect the internal organization of care (in this specific case, of diagnosis of smear-negative cases) rather than a patient's ability to appear at a health care unit.

In summary, 62% of patients started treatment after 60 days and half after 90 days. In our setting, delays do not appear to be caused by difficulty of access, age, sex or alcoholism. In this urban setting, delays seem to be linked to unemployment and the general attitude towards health, and reflect the way the health services are organized.

## Conclusion

There is a need to monitor the impact of the FHP and modify staff training to include active identification of symptoms that may indicate tuberculosis, and to increase population awareness of tuberculosis symptoms (building on the fact that the population already recognizes weight loss), with emphasis on developing general health awareness. A review of practices in the districts with short and long delays, to improve health services organization, should also be carried out.

## List of abbreviations

TCP Tuberculosis control Programme

FHP Family Health Programme

TB Tuberculosis

HIV Human Immunodeficiency virus

## Competing interests

The author(s) declare that they have no competing interests.

## Authors' contributions

MDPS, OMSD, ARLC, NLCLS, AMK and URM trained the field workers and participated in data entry and analysis. MDPS, MFPMA, CB, RAAX, URM, WVS and LCR interpreted the results and drafted the manuscript. MFPMA, RAAX, MDPS, LCR conceived of the study, and participated in its design and coordination. All authors read and approved the final manuscript.

## Pre-publication history

The pre-publication history for this paper can be accessed here:


